# Human bone marrow stromal cells: the impact of anticoagulants on stem cell properties

**DOI:** 10.3389/fcell.2023.1255823

**Published:** 2023-09-18

**Authors:** Michaela Ferencakova, Andrea Benova, Ivan Raska, Pavel Abaffy, Radek Sindelka, Martina Dzubanova, Eliska Pospisilova, Katarina Kolostova, Tomas Cajka, Ales Paclik, Vit Zikan, Michaela Tencerova

**Affiliations:** ^1^ Laboratory of Molecular Physiology of Bone, Institute of Physiology of the Czech Academy of Sciences, Prague, Czechia; ^2^ Faculty of Science, Charles University, Prague, Czechia; ^3^ Third Department of Medicine-Department of Endocrinology and Metabolism, First Faculty of Medicine, General University Hospital in Prague, Charles University, Prague, Czechia; ^4^ Laboratory of Gene Expression, Institute of Biotechnology of the Czech Academy of Sciences, Vestec, Czechia; ^5^ Laboratory of Personalized Medicine, Oncology Clinic, University Hospital Kralovske Vinohrady, Prague, Czechia; ^6^ Laboratory of Translational Metabolism, Institute of Physiology of the Czech Academy of Sciences, Prague, Czechia; ^7^ First Department of Surgery, First Faculty of Medicine, General University Hospital in Prague, Charles University, Prague, Czechia

**Keywords:** human bone marrow stromal cells, anticoagulants, cultivation, stem cell characteristics, differentiation potential

## Abstract

**Background:** Bone marrow stromal cells (BMSCs) are the source of multipotent stem cells, which are important for regenerative medicine and diagnostic purposes. The isolation of human BMSCs from the bone marrow (BM) cavity using BM aspiration applies the method with collection into tubes containing anticoagulants. Interactions with anticoagulants may affect the characteristics and composition of isolated BMSCs in the culture. Thus, we investigated how anticoagulants in isolation procedures and cultivation affect BMSC molecular characteristics.

**Methods:** BM donors (age: 48–85 years) were recruited from the hematology clinic. BM aspirates were obtained from the iliac crest and divided into tubes coated with ethylenediaminetetraacetic acid (EDTA) or heparin anticoagulants. Isolated BMSCs were analyzed by flow cytometry and RNA-seq analysis. Further cellular and molecular characterizations of BMSCs including CFU, proliferation and differentiation assays, cytometry, bioenergetic assays, metabolomics, immunostaining, and RT-qPCR were performed.

**Results:** The paired samples of isolated BMSCs obtained from the same patient showed increased cellular yield in heparin vs. EDTA samples, accompanied by the increased number of CFU colonies. However, no significant changes in molecular characteristics were found between heparin- and EDTA-isolated BMSCs. On the other hand, RNA-seq analysis revealed an increased expression of genes involved in nucleotide metabolism and cellular metabolism in cultivated vs. non-cultivated BMSCs regardless of the anticoagulant, while genes involved in inflammation and chromatin remodeling were decreased in cultivated vs. non-cultivated BMSCs.

**Conclusion:** The type of anticoagulant in BMSC isolation did not have a significant impact on molecular characteristics and cellular composition, while *in vitro* cultivation caused the major change in the transcriptomics of BMSCs, which is important for future protocols using BMSCs in regenerative medicine and clinics.

## Introduction

Bone marrow stromal cells (BMSCs) are a source of multipotent stem cells important for tissue engineering, regenerative medicine, and the diagnosis of bone and cancer diseases in patients. The isolation procedure of BMSCs from the bone marrow (BM) cavity using BM aspiration includes the process of collection into blood tubes containing anticoagulants to avoid coagulation of BM aspirate. BMSCs are known to go through the selection by an adherence to the plastic and creating colony-forming unit-fibroblast (CFU-f) in order to expand *in vitro* (Bianco et al., 2008). Anticoagulants act as calcium-binding agents, and thus, they may affect the cellular and molecular characteristics of isolated cells (Sadagopan et al., 2003). However, we do not know how exactly the processing of BM samples and interactions with anticoagulants may affect stem cell characteristics and composition of isolated BMSCs in the culture.

Anticoagulants used for blood collection have different modes of actions. Heparin is an anti-thrombin activator, and ethylenediaminetetraacetic acid (EDTA) is a metal ion chelator. Heparin prevents the conversion of fibrinogen into fibrin. However, heparin can interfere with various clinical assays, as documented in several studies reporting an influence on sample preparation and cell marker expression in heparin-collected blood samples (Walencik and Witeska, 2007; Freitas et al., 2008; Ibeagha-Awemu et al., 2012). EDTA chelates free calcium ions (Ca^2+^) in plasma. Thus, EDTA acts as an anticoagulant and chelating agent, interfering with calcium assays and clot generation (Bowen and Remaley, 2014). Furthermore, EDTA binds metallic ion europium (important for immunoassay reagent), zinc, and magnesium, which are enzyme cofactors for immunoassay reagents such as alkaline phosphatase (ALP) (Tate and Ward, 2004). Thus, it is important to consider how the blood and BM samples need to be collected for the subsequent assays and analyses. Data regarding the variation introduced by clinical sample collection processes are needed to avoid introducing artifact biases. Previous studies have already documented that hypothermia, hypoxia, analgesics, and needle passage affect cellular and molecular characteristics of mesenchymal stem cells (MSCs) (Galeano-Garces et al., 2017; Wu et al., 2018; Kubrova et al., 2020). Thus, the detailed information about the BMSC isolation procedure should be included in all protocols to maintain consistency in the clinical use of stem cells as it was recommended by Bone Marrow Adipose Society (BMAS) position paper (Lucas et al., 2021).

Generally, samples collected in EDTA-coated tubes are preferred for hematology testing, as it has been demonstrated to have minimal influence on cell morphology (Banfi et al., 2007). Similarly, heparin does not cause a change in cell size, although its ability to induce leukocyte agglutination and disintegration after several hours is often described (Ladinsky and Westring, 1967). However, clinicians and scientists have different preferences for using different types of blood collection tubes when it comes to the collection of BM aspirates. Therefore, it is important to evaluate whether the type of anticoagulant may change the properties and composition of isolated BMSCs.

Proteomic studies showed that heparinized plasma causes non-specific protein binding, which influences peptide separation and mass spectrometry (Tammen et al., 2005). On the other hand, the ability of heparin to interact with many proteins makes it a potential therapeutic agent beyond its use as an anti-thrombotic reagent (Lane and Adams, 1993). Heparin’s high affinity for proteins has resulted in its application in cell culture to enhance the desirable activity of critical extracellular biomolecules used as supplements to expand human stem cells. Heparin has already been reported to promote both WNT and FGF signaling in human embryonic stem cells, thereby increasing their proliferation (Furue et al., 2008; Sasaki et al., 2008). Similarly, heparin has been shown to enhance Wnt-induced differentiation signals in osteogenic cells (Ling et al., 2010), further highlighting its diverse effects and supporting osteogenic properties of stem cells. Tissue culture surfaces coated with glycosaminoglycans such as heparin support the greater proliferation of MSCs (Uygun et al., 2009; Ratanavaraporn and Tabata, 2012). Heparin-functionalized hydrogels and heparinized nanoparticles have also been developed to support the viability and differentiation of human MSCs (Benoit et al., 2007; Na et al., 2007).

However, it has not been reported whether the collection of BM aspirates in tubes coated with EDTA or heparin anticoagulants may change the yield and cellular properties of isolated BMSCs. Therefore, we aimed to test the hypothesis that human BMSCs (hBMSCs) isolated from BM aspirates collected in heparin- or EDTA-coated blood tubes do not differ in their cellular and molecular characteristics.

## Materials and methods

### Human subjects

Participants, including six men and six women [age 48–85 years (average 70 years)], were recruited from the hematology clinic. All participants signed informed consent prior to participation in the study. Ethical approval was obtained from the Ethic Committee (IORG0002175, IRB00002705, and FWA00029052) of the General University Hospital in Prague (156/22 S-IV). Samples were collected in accordance with the Declaration of Helsinki.

### Isolation and culture of hBMSCs from BM aspirates

BM samples were obtained by aspiration of 2–4 mL from the iliac crest after administration of the local anesthetic (lidocaine, 10 mg/mL) into a 20 mL syringe and mixed 1:1 with heparin (cat. number 0093746) (100 U/mL) or collected in EDTA-coated tubes (BD Vacutainer K2E EDTA; cat. number 367864). Low-density mononuclear cells were isolated through centrifugation using a Lymphoprep density gradient (density = 1.077 ± 0.001 g/mL) and then selected through the process of plastic adherence. Cells were then cultured at 1 × 10^5^ cells/cm^2^ (1 × 10^6^ cells per chamber slide) in minimal essential media (MEM, Gibco) containing 10% fetal bovine serum (FBS, Gibco) and 1% penicilin/streptomycin (P/S, Gibco), incubated at 5% CO_2_ at 37°C, and then, nourished by completely changing the medium once a week along with the passage at 70% confluence. Cultivated cells were sub-cultured and further studied under differentiation conditions to induce adipogenesis and osteogenesis, respectively. Due to the limited number of hBMSCs, it was not possible to test every donor sample by all testing methods. Specification of samples used in individual experiments is provided in the description of each experiment.

### CFU-f assay

After hBMSC isolation, cells from eight selected donors (donors 1, 2, 4, 5, 6, 7, 8, and 9) were seeded for CFU-f 0.5 mil. cells/25 cm^2^ flask and cultivated in growth media. After 14 days in culture, colonies displaying more than 50 cells were counted using crystal violet staining (Merck).

### Short-time proliferation assay

Isolated hBMSCs from six selected donors (donors 4, 5, 6, 7, 8, and 9) were plated in the 12-well plate in triplicate at a density of 5,000 cells/well in a standard growth medium. Cell number was evaluated after 1, 3, 6, 9, and 12 days. Cells were washed with PBS, detached by trypsinization, and then, manually counted using the Bürker–Türk counting chamber.

### *In vitro* differentiation of hBMSCs

Primary hBMSCs from two selected donors (donors 8 and 9) from passage 2 were used for analyzing their differentiation capacity as previously described (Tencerova et al., 2019).

### Osteoblast differentiation

hBMSCs were seeded at a density of 20,000 cells/cm^2^. When the cells reached 80% confluence, MEM (Gibco) supplemented with 10% FBS (Gibco) and 1% P/S (Gibco) was added to the control cells, and the rest of the cells were cultivated with osteoblast (OB) induction medium consisting of 10 mM β-glycerophosphate (Merck), 10 nM dexamethasone (Merck), 50 μg/ml vitamin C (Wako), and 10 nM vitamin D3 (Merck). The medium was changed every second day for 7 days (ALP activity) and 11 days (Alizarin Red S staining was provided).

### Alizarin Red S staining

Mineralization of cell matrix at day 11 was measured using Alizarin Red S staining. Cells were fixed with 70% ice-cold ethanol for minimum 1 h at −20°C after which Alizarin Red S solution (Merck) was added. The cells were stained for 10 min at room temperature (RT). Excess dye was washed with distilled water followed by PBS.

### ALP activity assay

ALP activity and cell viability assay were quantified at day 7 of OB differentiation in order to normalize the ALP activity data to the number of viable cells. Cell viability assay was performed using CellTiter-Blue Assay Reagent (Promega) at fluorescence intensity (579_Ex_/584_Em_). ALP activity was determined by absorbance at 405 nm using p-nitrophenyl phosphate (Sigma-Aldrich) as a substrate.

### Adipocyte differentiation

Cells were plated at a density of 30,000 cells/cm^2^. For adipocyte (AD) differentiation, DMEM (Gibco) was used, containing 10% FBS (Gibco), 1% P/S (Gibco), 100 nM dexamethasone (Merck), 450 μM 3-isobutyl-1-methylxanthine (IBMX) (Merck), 1 µM BRL (Merck), and 3 μg/mL insulin (Merck). The medium was changed every third day for 10 days (followed by Oil Red O and Nile Red staining).

### Oil Red O staining

At day 10 of differentiation, cells were rinsed with PBS and fixed in 4% paraformaldehyde (Merck) for 10 min at room temperature. After fixation, cells were briefly rinsed with 3% isopropanol solution (Merck), and lipid droplets were stained with Oil Red O solution (Merck) for 1 h at room temperature.

### Nile Red staining

Nile Red Staining was performed as previously described (Benova et al., 2022). Cells were cultured in polystyrene flat-bottom 96-well tissue culture-treated black microplates (BRANDplates^®^, cellGrade™, Brand, DE). Nile Red dye (Merck) working solution was prepared from a stock solution of 1 mg/ml. Cells were washed with PBS (Gibco). Dye was added directly to the cells (5 μg/ml in PBS), incubated for 10 min at room temperature in the dark, and then, washed twice with PBS. The fluorescent signal was measured using a Cytation 3 cell imaging multimode plate reader (BioTek) using an excitation of 485 nm and an emission of 572 nm. The fluorescent signal of Nile Red stain was normalized to cell viability signal measured by Cell Titer-Blue Assay Reagent (Promega) mentioned previously.

### Fluorescence microscopy

The cells obtained from two selected donors (donors 8 and 9) were stained by vital fluorescence stains [NucBlue™ and MitoTracker™ Red CM-H2Xros (Thermo Fisher Scientific, Waltham, MA, United States)]. The stains were added according to the manufacturer’s recommendations directly into the culturing flask and incubated for 30 min. Furthermore, the medium was changed to fresh MEM supplemented with 10% FBS (Gibco) + 1% P/S + 1% GlutaMax + 1% MEM non-essential amino acid solution (NEAA) + 1% sodium pyruvate. Cells were examined by fluorescence microscopy in two steps: 1) screening at ×20 magnification to locate the cells and 2) observation at ×40/×60 magnification for detailed cytomorphological analysis.

### Isolation of RNA and RT-qPCR

Total RNA obtained from two selected donors (donors 8 and 9) was isolated using TRI Reagent (Merck), and RNA concentration was measured using NanoDrop spectrophotometer. cDNA synthesis was performed from 1 µg of total RNA using the High Capacity cDNA Reverse Transcription Kit (Thermo Fisher) according to the manufacturer’s protocol. Quantitative real-time PCR was performed using the LightCycler^®^ 480 SYBR Green I Master (Roche) with specific primers listed in Supplementary Table S1. RT-qPCR data were normalized to β-actin housekeeping gene expression.

### RNA sequencing of hBMSCs

Total RNA obtained from two selected donors (donors 10 and 11) was isolated, and RNA-seq was performed on hBMSCs obtained from heparin- and EDTA-coated tubes. One set of samples was analyzed right after the isolation (non-cultivated hBMSCs), and a second set was analyzed 13 days after *in vitro* cultivation until cells were confluent (cultivated hBMSCs).

The quality of RNA was measured using the Fragment Analyzer RNA Kit (Agilent Technologies, DNF-471), and samples with RQN value > 7 were used for library preparation. RNA sequencing libraries were prepared from 50 ng of total RNA using QuantSeq 3’mRNA-Seq Library Prep FWD for Illumina (Lexogen, 015.96) with the UMI Second Stranded Synthesis Module for QuantSeq FWD (Lexogen; 081.96) according to the manufacturer’s protocol. Final libraries were pooled together and sequenced on Illumina NextSeq^®^ 500/550 (High Output Kit v2.5; 20024906) with 86 bp single-end reads. Approximately 20 M reads per sample were obtained. First, the quality of raw reads was checked using FastQC v0.11.9, and the potential contamination was screened using FastQ_Screen v0.11.1 (Wingett and Andrews, 2018). Then, the 6 bp-long UMIs followed by “TATA” spacer were added to reads name using umi_tools extract function (https://genome.cshlp.org/content/early/2017/01/18/gr.209601.116.abstract). The low-quality reads and adaptor sequences were removed using TrimmomaticSE v0.36 with parameters “HEADCROP:4 ILLUMINACLIP: Lexogen_quantseq.fa:2:30:10 LEADING:3 TRAILING:3 SLIDINGWINDOW:4:15 MINLEN:36.” Ribosomal and mitochondrial reads were removed using SortMeRNA v2.1b (Kopylova et al., 2012). BAM files with alignment were created with STAR v2.7.0f (https://pubmed.ncbi.nlm.nih.gov/23104886/) (reference *Homo sapiens* genome version GRCh38). Reads that were mapped to more than one place in the genome were not processed. The count tables were generated using the script HTseq-count v0.11.4 (https://academic.oup.com/bioinformatics/article/31/2/166/2366196) with the annotation version GRCh38.87 and parameter “-m union.” ENSEMBL-IDs were used as identifiers of transcripts.

The counted data were analyzed using the R package DESeq2 v1.34.0 (Love et al., 2014). Rlog-transformed data were processed by the principal component analysis. Differentially expressed genes (DEGs) were identified by the command “DESeq” with default parameters. ENSEMBL-IDs were converted into gene symbol using the org.Hs.eg.db v3.14.0 database (Carlson, 2019). Genes with baseMean > 20 and adjusted *p*-value < 0.1 were considered DEGs; log_2_FoldChange > 0.6 for upregulated DEGs and log_2_FoldChange < 0.6 for downregulated DEGs. Functional Gene Ontology overrepresentation analysis (ORA) was conducted using the web tool WebGestalt 2019 (https://academic.oup.com/nar/article/47/W1/W199/5494758?login=false) using these parameters: organism—*H. Sapiens*, method of interest—ORA, functional database—Gene Ontology, biological process noRedundant, selected gene ID type—Ensembl gene ID, select reference set—genome, minimum number of genes for a category—five, maximum number of genes for a category—2000, multiple test adjustment—BH, significance level—TOP 10, number of categories expected from set cover—10, number of categories visualized in the report—40, and color in DAG—continuous.

### Flow cytometry

hBMSCs obtained from two randomly selected donors (donors 8 and 11) were immunophenotyped using a panel of cell surface markers (Supplementary Table S2). Adherent cells were released from flasks using 0.05% trypsin/EDTA and incubated with the Fc-human blocking reagent followed by incubation with pre-conjugated antibodies for specific markers according to the manufacturer’s recommendations. The flow cytometry was performed by LSRII (BD Biosciences) and analyzed by FlowJo 10.8.0 analysis software.

### Bioenergetic analysis

Parallel measurement of the oxygen consumption rate (OCR) and extracellular acidification rate (ECAR) was performed using the Seahorse XFe24 Analyzer (Seahorse, Agilent). Primary hBMSCs obtained from two selected donors (donors 8 and 9) were seeded in the 24-well Agilent Seahorse XF cell culture microplate in 5-plicates at a density of 20,000 cells/well in the growth medium the day prior to the analysis. The next day, all wells were washed with 1 mL of Dulbecco’s modified Eagle’s medium (D5030, Merck) supplemented with 10 mM glucose, 4 mM glutamine, and 2 mM pyruvate (pH 7.4; 37°C); 500 µL of the same medium was pipetted, and the microplate was incubated at 37°C for 30 min. Meanwhile, the XFe24 sensor cartridge was prepared by injection of substrates according to the protocol to measure metabolic rates with endogenous substrates (basal) and after subsequent additions with final concentrations of 10 mM glucose (Merck), 1 µM oligomycin (Oligo) (Merck), 2 µM carbonyl cyanide-4-(trifluoromethoxy) phenylhydrazone (FCCP) (Merck), and the mixture of inhibitors of 1 µM rotenone (Rot) (Merck), 1 μg/mL of antimycin A (AA) (Merck), and 100 mM 2-deoxyglucose (2DG) (Merck) (2DG+AA+Rot). The Seahorse data were analyzed using Wave Software 2.6.1 (Agilent). The data were normalized by the cell number determined by Hoechst 33342 staining of cell nuclei (final concentration 5 μg/mL) (Thermo Fisher), which was performed immediately after the measurement using the Cytation 3 cell imaging multimode plate reader (BioTek) and processed using Gen5 software (BioTek).

### Lipidomics and metabolomics

Global lipidomic and metabolomic profiling of human BM plasma samples was conducted using a combined untargeted and targeted workflow for lipidome, metabolome, and exposome analysis (LIMeX) (Janovska et al., 2020; Tsugawa et al., 2020; Sistilli et al., 2021) with some modifications. Extraction was performed using a biphasic solvent system of cold methanol, methyl *tert*-butyl ether (MTBE), and 10% methanol. Four different liquid chromatography–mass spectrometry (LC–MS) platforms were used for profiling: 1) lipidomics of complex lipids using reversed-phase liquid chromatography with mass spectrometry (RPLC–MS) in the positive ion mode, 2) lipidomics of complex lipids in RPLC–MS in the negative ion mode, 3) metabolomics of polar metabolites using hydrophilic interaction chromatography with mass spectrometry (HILIC–MS) in the positive ion mode, and 4) metabolomics of polar metabolites using RPLC–MS in the negative ion mode. Sample preparation and data processing were performed as described in the previous publication (Benova et al., 2022). The raw metabolomic data with peak height intensities of annotated metabolites are presented in Supplementary Table S3.

### Statistical analysis

The statistical significance of the differences between the experimental groups (heparin vs. EDTA) was determined by paired Student’s t-test using GraphPad Prism 9.3.1 software. Data are presented as means ± SEM. *p*-value < 0.05 was considered statistically significant. Negative binomial generalized linear model fitting and Wald statistics with the Benjamini–Hochberg test were used for bulk RNA sequencing. Metabolomics and lipidomics data were processed using MetaboAnalyst (Pang et al., 2022). All the statistical details of experiments can be found in the figure legends.

## Results

### Effect of heparin and EDTA anticoagulants on the cellular characteristics of hBMSCs

To investigate whether the collection of human BM aspirates in tubes coated with heparin or EDTA anticoagulant affects the cellular properties of isolated hBMSCs, we enrolled 12 random donors (6M/6F) from the hematology clinic. These BM samples were divided into heparin-coated and EDTA-coated blood tubes to obtain paired samples of BM aspirate for further cellular and molecular analyses of isolated hBMSCs. The basic characteristics of enrolled donors are summarized in Table 1.

**TABLE 1:** Basic clinical information about the donors of BM aspirates [*n* = 12 donors (6M/6F)].

Donor	Gender (M/F)	Age (years)	Pre-existing condition	Biopsy post-chemotherapy
1	M	77	Myelodysplastic syndrome	No
2	M	56	Acute myeloid leukemia	No
3	F	85	Marginal zone lymphoma	Yes
4	M	76	Follicular lymphoma	No
5	F	48	Multiple myeloma	Yes
6	F	77	Myelodysplastic syndrome	No
7	M	74	Refractory cytopenia with multilineage dysplasia	No
8	M	65	Multiple myeloma	Yes
9	F	69	Lymphoplasmacytic lymphoma	No
10	M	72	Diffuse large B-cell lymphoma	No
11	F	80	Mantle cell lymphoma	No
12	F	65	Follicular lymphoma	No

**FIGURE 1:**
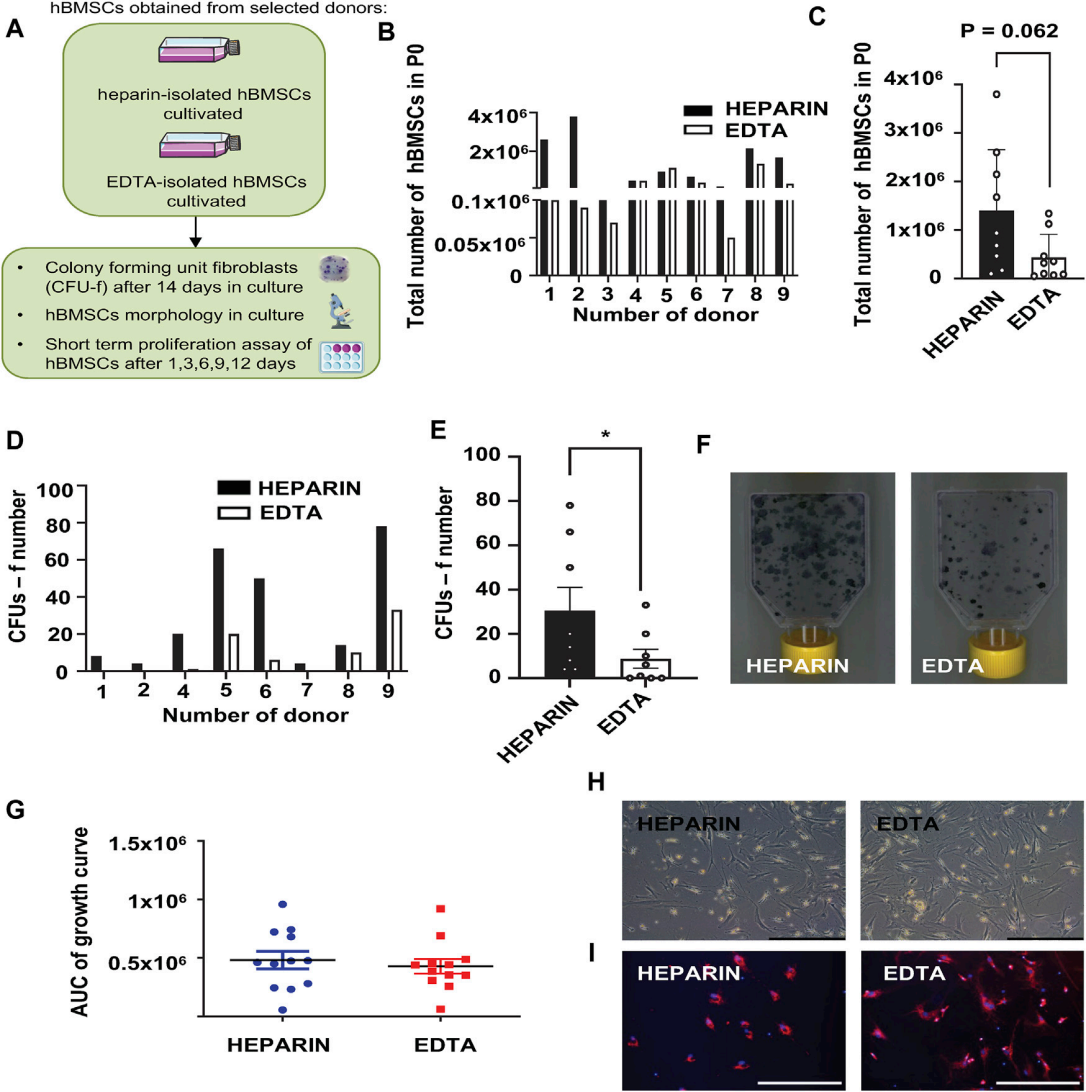
Effects of heparin and EDTA anticoagulants on cellular characteristics of hBMSCs. **(A)** Schematic of the experiment. **(B)** Total number of hBMSCs isolated from heparin- and EDTA-coated tubes in passage 0. Data are presented as individual values in heparin and EDTA samples from individual donors (*n* = 9). **(C)** Total number of hBMSCs isolated from heparin- and EDTA-coated tubes in passage 0. Data are presented as mean ± SEM (*n* = 9); (*p* > 0.05 and paired Student’s t-test). **(D)** Number of CFUs-f of hBMSCs isolated from heparin- and EDTA-coated tubes, seeded directly after isolation and evaluated after 14 days in growth media. Data are presented as individual values in heparin and EDTA samples from individual donors (*n* = 8). **(E)** Calculation of CFUs-f of hBMSCs isolated from heparin- and EDTA-coated tubes; data are presented as mean ± SEM (*n* = 8 per group); (*p < 0.05 and paired Student’s t-test). **(F)** Representative pictures of CFUs-f of hBMSCs obtained from heparin- and EDTA-coated tubes. **(G)** Short-term proliferation rate presented as the area under the curve (AUC) of primary hBMSCs isolated from heparin- and EDTA tubes calculated after 1, 3, 6, 9, and 12 days in culture after seeding. Data are presented as mean ± SEM (*n* = 12); (*p* > 0.05 and paired Student’s t-test). **(H)** Representative pictures of hBMSC morphology (scale bar 200 μm; ×10 magnification) in heparin and EDTA samples. **(I)** Representative fluorescent pictures of hBMSCs isolated from heparin and EDTA tubes stained using MitoTracker Red (red—mitochondrion) and NucBlue (blue—nucleus) (scale bar 200 μm; ×20 magnification).

The paired samples of isolated hBMSCs from heparin- and EDTA-coated tubes obtained from the same donors were analyzed for their basic cellular characteristics (Figure 1A; Table 2). While the yield of hBMSCs after isolation was not different between heparin- vs. EDTA-coated tubes, the yield of hBMSCs after passage 0 (when they reach the confluence in a flask) was higher in the samples obtained from heparin-coated tubes compared to EDTA-coated tubes, with a trend toward significance (*p* = 0.062) (Figures 1B, C). Furthermore, the number of CFU-f, an *in vitro* surrogate marker of the stem cell potency of hBMSCs, was higher in all heparin-isolated hBMSCs in comparison to EDTA-isolated hBMSCs (*p* = 0.017) (Figures 1D–F), which might suggest a presence of more potent stem cell populations in heparin-isolated samples. On the other hand, the short-term proliferation rate of hBMSCs was not affected by the type of anticoagulants used to prepare BM aspirates (Figure 1G). In addition, microscopic analysis of cell morphology in the cultivated hBMSCs did not reveal any significant changes between heparin- and EDTA-isolated hBMSCs (Figure 1H; Supplementary Figure S1A). Moreover, using fluorescent markers for the nucleus (NucBlue ™) and active mitochondria (MitoTracker ™ Red CM-H2Xros) did not show any profound differences between heparin- and EDTA-isolated hBMSCs (Figure 1I).

**TABLE 2:** Cellular characteristics of hBMSCs [*n* = 12 donors (6M/6F)].

Donor	Coated blood tubes	Volume of bone marrow (mL)	Number of mononuclear cells (×10^6^)/mL of bone marrow	Time in culture p0 (day)	Total number of hBMSCs (×10^6^)—passage 0	Total number of hBMSCs (×10^6^)—passage 1	CFU-F/0.5 mil
1	Heparin	4	21.3	18	2.6	0.73	8
	EDTA	1.5	10.2	28	0.1	0.15	0
2	Heparin	7	23.6	24	3.8	1.5	4
	EDTA	1.5	7.4	31	0.09	0.08	0
3	Heparin	2	1.4	30	0.1	0.075	-----
	EDTA	1	7.7	32	0.07	0.015	-----
4	Heparin	4	3.9	30	0.47	1.13	20
	EDTA	1	15.3	34	0.46	0.41	1
5	Heparin	4	3.6	16	0.94	0.98	66
	EDTA	2	9.6	23	1.13	0.98	20
6	Heparin	3	6.2	23	0.68	0.44	50
	EDTA	2	4.2	34	0.36	0.5	6
7	Heparin	4	0.9	33	0.17	0.4	4
	EDTA	3	2.4	33	0.05	0.22	0
8	Heparin	15	5.6	24	2.15	1.6	14
	EDTA	5.5	10.6	24	1.34	1.48	10
9	Heparin	11	2.2	15	1.68	1.64	78
	EDTA	5.5	2.2	20	0.32	0.45	33
10	Heparin	5.5	2.1	24	0.55	-----	-----
	EDTA	5	1	-----	-----	-----	-----
11	Heparin	5	2.5	18	0.83	-----	-----
	EDTA	2.5	2	-----	-----	-----	-----
12	Heparin	5.5	5.2	18	1.49	-----	-----
	EDTA	1.8	3	-----	-----	-----	-----

### Impact of cultivation on the transcriptomic profile of hBMSCs vs. use of different anticoagulants during BM collection

**FIGURE 2:**
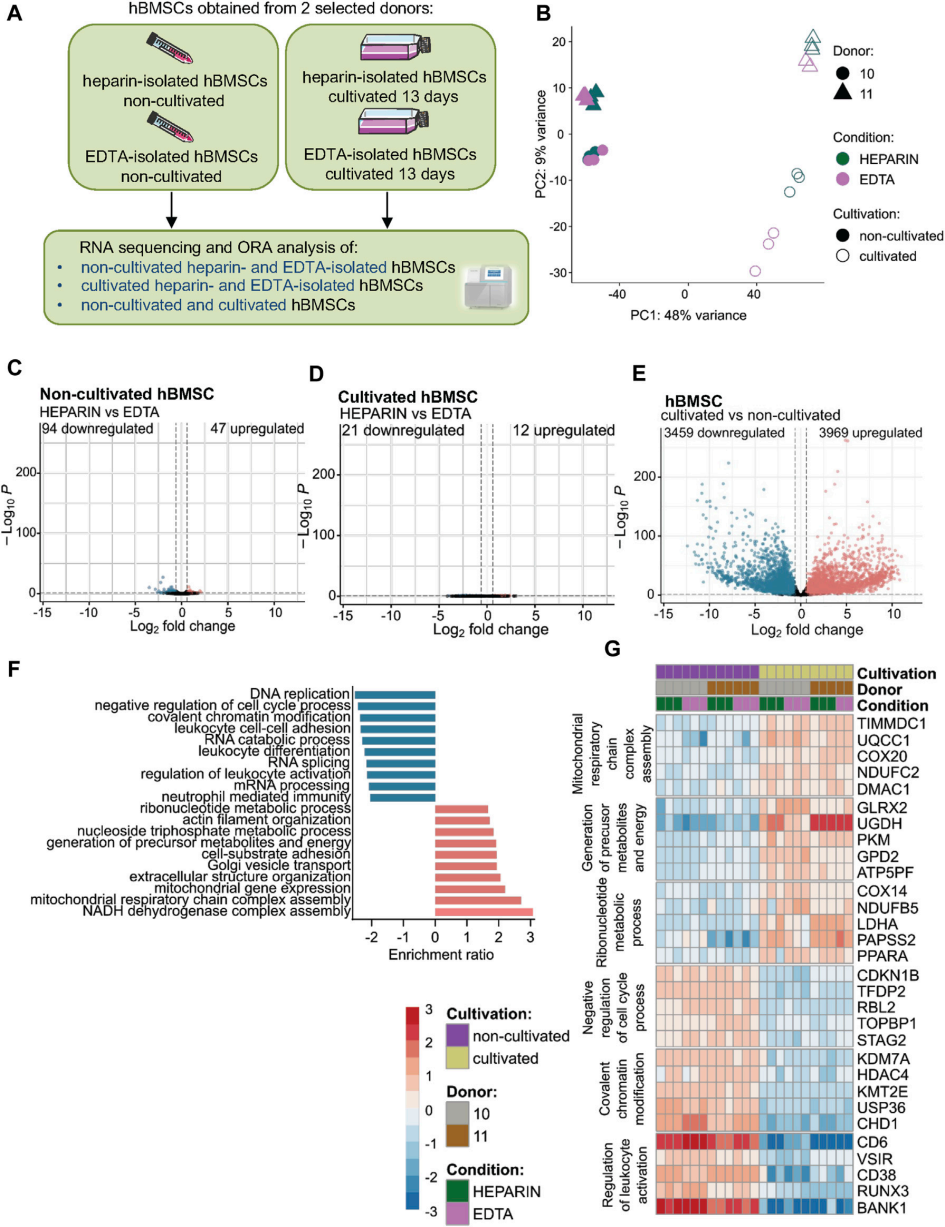
Effects of heparin and EDTA anticoagulants and cultivation on the transcriptomic profile of non-cultivated and cultivated hBMSCs. hBMSC cultures were obtained from two selected donors (donors 10 and 11). RNA was isolated, and RNA-seq was performed on non-cultivated and cultivated hBMSCs cells obtained from heparin- and EDTA-coated tubes. **(A)** Schematic of the experiment. **(B)** The principal component analysis plot shows the percentage of variation between non-cultivated and cultivated hBMSCs isolated from heparin- and EDTA-coated tubes. The first two principal components are plotted and colored according to donor, condition, and cultivation. PCA was performed using log-transformed data from all analyzed samples and the top 500 most variable genes. The percentage of variation accounted for each principal component is shown with the axis label. **(C–E)** Volcano plots showing downregulated and upregulated gene levels in **(C)** non-cultivated hBMSCs isolated from heparin-coated and EDTA-coated tubes, **(D)** in cultivated hBMSCs isolated from heparin- and EDTA-coated tubes, and **(E)** in the cultivated hBMSCs compared to non-cultivated hBMSCs (adjusted *p*-value < 0.1; absolute log_2_ fold change > 0.6). **(F)** ORA analysis was conducted using the web tool WebGestalt 2019. The enrichment ratio bar chart shows downregulated (blue) and upregulated (red) top 10 set of genes involved in the molecular pathways in cultivated hBMSCs compared to non-cultivated hBMSCs. **(G)** The heat map was constructed with the log-transformed count data subtracted by the average value across each gene.

To further understand the impact of used anticoagulants on the molecular characteristics of isolated hBMSCs, we performed RNA sequencing (RNA-seq) of paired samples of heparin- and EDTA-isolated hBMSCs. In addition, we investigated whether hBMSC expansion in the culture changes their transcriptomic profiles depending on the type of anticoagulants used in the isolation process (Figure 2A).

Principal component analysis (PCA) showed minimal separation between paired hBMSCs from heparin and EDTA samples, which were analyzed directly after isolation (non-cultivated) (Figure 2B). From around 10,000 genes identified in non-cultivated hBMSCs, comparative analysis revealed only 141 significant DEGs (Figure 2C; Supplementary Table S4), from which 94 were downregulated and 47 were upregulated in heparin-isolated BMSCs compared to EDTA-isolated BMSCs. This number of differently expressed genes between non-cultivated heparin-isolated vs. EDTA-isolated hBMSCs suggests no significant impact of the used anticoagulants on the transcriptomic profile of hBMSCs, if measured directly after the isolation process.

Similarly, minimal changes were observed in paired samples of hBMSCs cultivated in growth media after isolation from heparin-coated and EDTA-coated tubes. From the total of 11,407 analyzed genes, we found only 12 upregulated and 21 downregulated genes in cultivated hBMSCs between heparin and EDTA samples (Figure 2D), suggesting that used anticoagulants did not show any significant differences between the transcriptome of cultivated hBMSCs obtained from heparin- and EDTA-coated tubes.

On the other hand, we found that cultivation had a much bigger effect on molecular characteristics of hBMSCs compared to used anticoagulants in the isolation procedure. The PCA plot of all cultivated and non-cultivated hBMSCs (Figure 2B; Supplementary Table S4) showed a clear separation without differences between the types of anticoagulants. The volcano plot revealed 11,560 significant detect genes, where 3,969 were upregulated and 3,459 downregulated in cultivated compared to non-cultivated hBMSCs (adjusted *p*-value < 0.100 and the absolute value of log2 fold change > 0.6) (Figure 2E; Supplementary Table S4). In order to elucidate the functionality of differentially expressed genes, we performed functional Gene Ontology overrepresentation analysis. Genes that exhibited higher expression with cultivation compared to non-cultivated cells (Figures 2F, G) were annotated in the GEO category for mitochondrial respiratory chain complex assembly and mitochondrial gene expression (TIMMDC1—translocase of inner mitochondrial membrane domain containing 1 and UQCC1—ubiquinol-cytochrome c reductase complex assembly factor 1), generation of precursor metabolites, and energy (GLRX2—glutaredoxin 2 and UGDH—UDP-glucose 6-dehydrogenase). Transcripts involved in the ribonucleotide metabolic process (COX14—cytochrome c oxidase assembly factor COX14 and NDUFB5—NADH:ubiquinone oxidoreductase 5) were also enriched in this subset. The remodeling of the mitochondrial and metabolic infrastructure matches the evolving bioenergetic requirements, as cells have a high energy demand. However, the genes involved in cell proliferation (e.g., CCNA2, CCNB1, and MKI67) (Supplementary Figure S2A) were not differently expressed between non-cultured and cultured cells. The genes that were downregulated with the *in vitro* cultivation (Figures 2F, G) are related to decreased inhibitory factors on cell cycle (CDKN1B—cyclin-dependent kinase inhibitor 1B and TFDP2—transcription factor Dp-2), chromatin remodeling (KDM7A—lysine demethylase 7A and HDAC4—histone deacetylase 4), and decreased inflammatory properties of the cells (CD6—cluster of differentiation 6; VSIR—V-set immunoregulatory receptor; and CD38—cluster of differentiation 38) suggesting the maintenance of stable expression states of transcription factor genes, the control of stem cell activity, higher proliferation, and active metabolic status of the stem cells.

### Effect of heparin and EDTA anticoagulants and subsequent cultivation on the cell surface markers of hBMSCs

**FIGURE 3:**
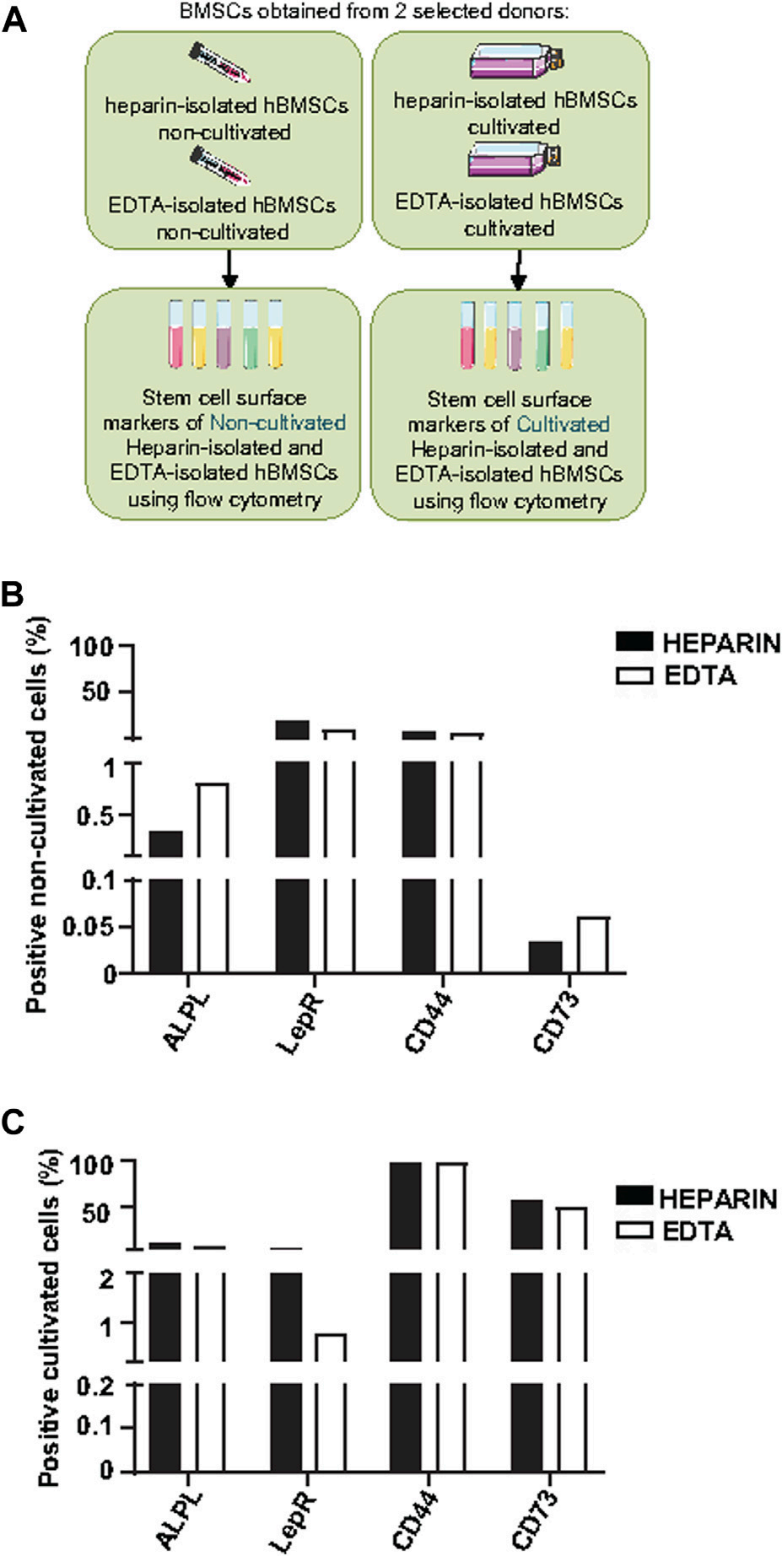
Effects of heparin and EDTA anticoagulants and cultivation on the cell surface markers of non-cultivated and cultivated human BMSCs. **(A)** Schematic of the experiment. **(B)** Screening of stem cell surface markers using flow cytometry in non-cultivated hBMSCs obtained directly after isolation from heparin-coated and EDTA-coated tubes from one selected donor (donor 8). Data are presented as a comparison of the percentage of positive cells between the heparin and EDTA experimental groups from one selected donor. **(C)** Screening of stem cell surface markers using flow cytometry in cultivated hBMSCs isolated from heparin- and EDTA-coated tubes from one selected donor (donor 11). Data are presented as a comparison of the percentage of the positive cells between heparin and EDTA experimental groups from one selected donor.

Because of differences in the BMSC yield and number of CFUs-f of cultivated heparin- and EDTA-isolated hBMSCs, we further analyzed the expression profile of cell surface markers in non-cultivated and cultivated hBMSCs obtained from heparin- and EDTA-coated tubes (Figure 3A). Using the panel of used stem cell markers (ALPL, LepR, CD44, and CD73) (Supplementary Figure S2A), no changes were found between non-cultivated hBMSCs obtained from heparin- and EDTA-coated tubes (Figure 3B; Table 3), and the same was observed in cultivated hBMSCs (Figure 3C; Table 4). The presence of cell surface markers (CD44 and CD73) differed between non-cultivated and cultivated hBMSCs, but they were not affected by the type of anticoagulants used in the isolation process. These data were also confirmed with RNA-seq analysis (Supplementary Figures S3A, B).

**TABLE 3:** Screening of stem cell surface marker expression measured by flow cytometry in non-cultivated hBMSCs obtained directly after isolation (*n* = 3).

Donor	Coated blood tube	ALP (%)	LepR (%)	CD44 (%)	CD73 (%)
10	Heparin	0.33	17.6	12.7	0.11
	EDTA	0.71	28.3	9.2	0
11	Heparin	0.38	9.79	5.32	0.03
	EDTA	0.5	11.5	8.98	0.06
12	Heparin	0.4	8.61	11.1	0.06
	EDTA	0.23	5.47	9.39	0.01

**TABLE 4:** Screening of stem cell surface marker expression measured using flow cytometry in cultivated hBMSCs (*n* = 6).

Donor	Coated blood tube	ALP (%)	LepR (%)	CD44 (%)	CD73 (%)
4	Heparin	22.5	0.74	99.6	88.8
	EDTA	35.4	1.37	-----	-----
5	Heparin	4.91	0.34	99.9	85.2
	EDTA	10.1	-----	99.7	91.9
6	Heparin	13	-----	-----	79.8
	EDTA	11.3	1.55	-----	-----
7	Heparin	13.5	6.42	99.9	97.3
	EDTA	5.18	1.3	-----	-----
8	Heparin	11.1	2.43	97.3	69.2
	EDTA	6.44	0.59	99.2	65.1
9	Heparin	27	0.56	94.3	80.2
	EDTA	-----	-----	-----	-----

Thus, these data suggest that heparin and EDTA anticoagulants did not affect the composition of isolated hBMSCs based on the basic panel of stem cell surface markers, and differences were detected only between non-cultivated and cultivated hBMSCs.

### Effect of heparin and EDTA anticoagulants on the differentiation capacity and bioenergetic profiles of hBMSCs

**FIGURE 4:**
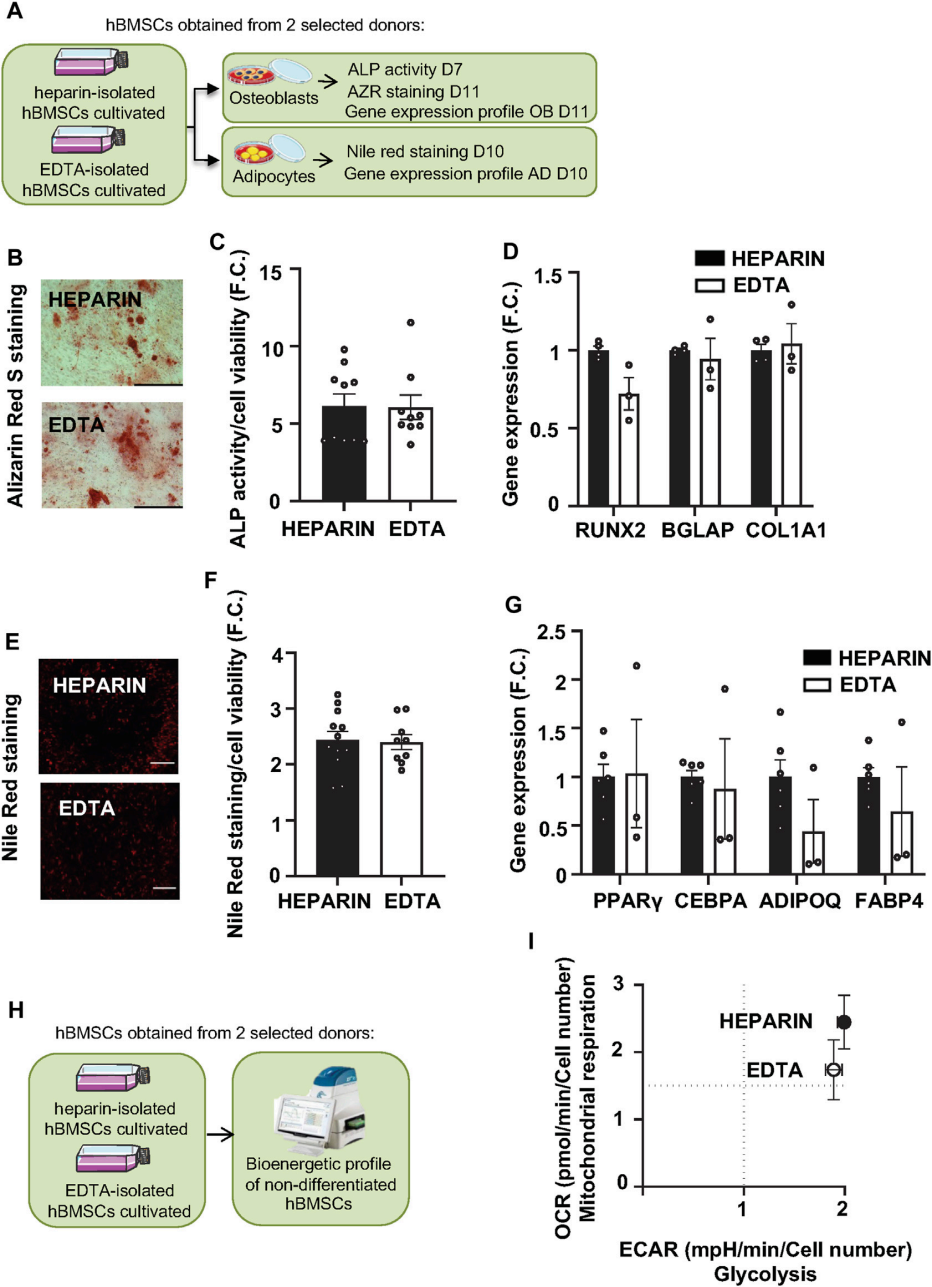
Effects of heparin and EDTA anticoagulants on the differentiation capacity and bioenergetic profile of hBMSCs. **(A)** Schematic of the experiment. **(B)** Representative pictures of Alizarin Red S (AZR) staining for calcified matrix mineralization of OB differentiated hBMSCs (scale bar 200 μm; ×10 magnification). **(C)** Measurement of ALP activity normalized to cell viability in OB differentiated hBMSCs in D7. Data are presented as mean fold change (F.C.) of ALP activity in non-differentiated cells vs. differentiated cells (OB) isolated from heparin and EDTA-coated tubes ± SEM (*n* = 2); (*p* > 0.05 and paired Student’s t-test). **(D)** Gene expression profile of osteoblastic markers (RUNX2, BGLAP, and COL1A1) in OB differentiated hBMSCs in D11. Data are presented as the mean fold change (F.C.) of gene expression normalized to hBMSCs from heparin group ± SEM (*n* = 2); (*p* > 0.05 and paired Student’s t-test). **(E)** Representative pictures of Nile Red staining of AD differentiated hBMSCs (scale bar 200 μm; ×4 magnification). **(F)** Quantification of Nile Red staining normalized to cell viability in AD differentiated hBMSCs in D10. Data are presented as mean F.C. of Nile red quantification in non-differentiated cells vs. differentiated cells (AD) isolated from heparin- and EDTA-coated tube ± SEM (*n* = 2); (*p* > 0.05 and paired Student’s t-test). **(G)** Gene expression profile of adipogenic genes (PPARγ, CEBPA, ADIPOQ, and FABP4) in AD differentiated hBMSCs in D10. Data are presented as mean F.C. of gene expression normalized to hBMSCs from heparin group ± SEM (*n* = 2); (*p* > 0.05 and paired Student’s t-test). **(H)** Schematic of the bioenergetic experiment. **(I)** XF Energy map visualizing the metabolic phenotype profile of cultivated hBMSCs. The energy map was calculated from the mean values of OCR and ECAR after adding glucose. Data are presented as mean ± SEM (*n* = 2 independent experiments with five replicates per sample).

In order to understand whether a type of anticoagulant may affect the differentiation capacity of cultivated hBMSCs, we tested the differentiation potential of heparin and EDTA hBMSCs in the osteogenic (OB) and adipogenic (AD) conditions *in vitro* (Figure 4A).

As previous findings documented that heparin stimulates osteogenic potential in mouse BMSCs (Ling et al., 2010), we hypothesized that heparin-isolated hBMSCs might have increased OB differentiation capacity *in vitro* compared to EDTA-isolated cells. However, Alizarin S (AZR) staining (Figure 4B), ALP activity (Figure 4C; Table 5), and the gene expression of OB markers (RUNX2, BGLAP, and COL1A1) in OB-differentiated hBMSCs (Figure 4D) did not show significant changes between heparin- and EDTA-isolated hBMSCs in different donors. In addition to AD differentiation of hBMSCs measured by Nile Red staining (Figures 4E, F), the gene expression of AD genes (PPARγ, CEBPA, ADIPOQ, and FABP4) (Figure 4G) also did not show any changes between heparin- and EDTA-isolated samples. Thus, these *in vitro* validations of hBMSC differentiation potential suggest no significant difference between samples obtained from heparin- and EDTA-coated tubes.

**TABLE 5:** Osteoblast differentiation potential of hBMSCs isolated from BM donors evaluated using quantification of alkaline phosphatase (ALP) activity represented as fold change (F.C.) over non-induced cells (day 7) (*n* = 9).

Donor	Coated blood tube	ALP activity (F.C.)
1	Heparin	6.77 ± 0.35
	EDTA	6.42 ± 0.56
2	Heparin	4.7 ± 0.38
	EDTA	2.73 ± 0.36
3	Heparin	2.82 ± 0.38
	EDTA	1.11 ± 0.07
4	Heparin	3.22 ± 0.7
	EDTA	3.48 ± 0.51
5	Heparin	2.36 ± 0.05
	EDTA	6.02 ± 0.5
6	Heparin	3.88 ± 0.41
	EDTA	6.53 ± 0.4
7	Heparin	4.52 ± 0.55
	EDTA	5.58 ± 0.74
8	Heparin	3.94 ± 0.04
	EDTA	4.75 ± 0.31
9	Heparin	8.35 ± 0.44
	EDTA	7.67 ± 1.41

To further investigate the effects of used anticoagulants on functional characteristics (cellular metabolism) of isolated hBMCSs, we performed bioenergetic profiling of heparin- and EDTA-isolated hBMSCs (from two selected donor samples) using Seahorse technology (Figure 4H). We obtained simultaneous measurements of mitochondrial function via OCR (Supplementary Figure S4A) and glycolysis via ECAR (Supplementary Figure S4B).

Glycolysis measurements did not reveal any significant changes in glycolytic capacity and glycolytic reserve between heparin and EDTA samples (Figure 4I). In addition, the measurements of cellular respiration also did not show any changes in maximal respiration induced by FCCP or spare capacity.

Overall, these findings assume that different types of anticoagulants in the BM collection procedure of hBMSCs do not influence the functional performance and quality of isolated hBMSCs. Thus, these types of samples might be comparable for future multicentric analysis.

### Effect of heparin and EDTA anticoagulants on the lipidome and metabolome of BM plasma

**FIGURE 5:**
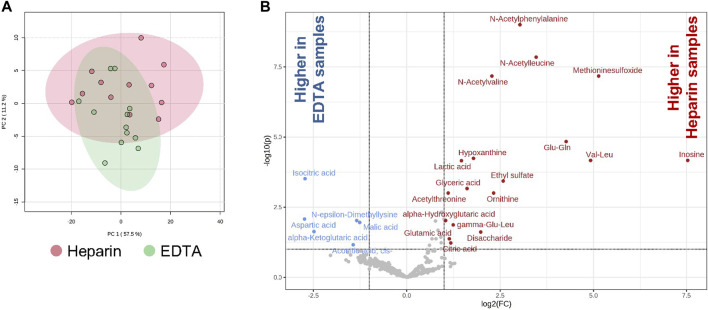
Effects of heparin and EDTA anticoagulants on lipidome and metabolome of BM plasma. **(A)** PCA of heparin and EDTA samples; **(B)** volcano plots showing the increase and decrease of polar metabolites in heparin and EDTA samples [adjusted (FDR) *p*-value < 0.1; absolute log2 fold change > 1].

In order to investigate the effects of heparin and EDTA on the lipidome and metabolome of BM plasma from the paired samples from the same patient, global lipidomic and metabolomic analyses were performed using a multiplatform liquid chromatography–mass spectrometry-based (LC–MS) approach. Overall, 470 polar metabolites and simple and complex lipids were annotated in these samples. For most annotated polar metabolites and lipids (95%), no differences between heparin and EDTA groups were observed (Figure 5A) except for a group of organic acids (isocitric acid, *cis*-aconitic acid, malic acid, aspartic acid, and α-ketoglutaric acid) that were increased in EDTA samples (*p* < 0.001). On the other hand, some organic acids (citric acid, lactic acid, glyceric acid, and α-hydroxyglutaric acid), amino acid derivatives (e.g., *N*-acetyl forms of alanine, leucine, valine, and phenylalanine), and dipeptides were increased in heparin samples (*p* < 0.001) (Figure 5B).

Thus, these data showed that the metabolomic profile in BM plasma is minimally affected between heparin- and EDTA-coated paired samples.

## Discussion

In this study, we evaluated the effect of anticoagulants used in the isolation procedure of hBMSCs on their cellular and molecular characteristics. The BM aspirates at the clinic usually come from heparin- or EDTA-coated blood tubes, and this aspect has never been assessed from the perspective of stem cell use in regenerative medicine. We also evaluated the impact of *in vitro* cultivation on hBMSC stem cell properties compared to freshly isolated cells (non-cultivated cells). While the type of anticoagulants in isolation protocol did not affect most of the cellular and molecular characteristics, the *in vitro* cultivation of hBMSCs in growth media caused a major change in the gene expression profile of hBMSCs, which is of importance for future protocols using hBMSCs and understanding the impact of the used isolation methods on downstream assays. This study brings an important knowledge for future clinical studies using a different source of hBMSCs compared to other published data.

Previous studies focused on studying the effects of anticoagulants on blood cell composition and the presence of anticoagulants in media to support stem cells, but not directly on the impact of different anticoagulants on hBMSC cellular and molecular characteristics (Furue et al., 2008; Sasaki et al., 2008; Baien et al., 2018; Gerber et al., 2020).

Hussen et al. (2022) reported that the composition of leukocytes differs between heparin- and EDTA-collected blood samples with heparin giving a lower proportion of specific blood cell populations. Furthermore, Baien et al. (2018) reported that K3EDTA blood resulted in higher purity of bovine granulocytes compared to lithium heparin blood, which also affected downstream functional assay on blood cells accompanied with increased CD11b expression and increased oxidative burst in heparin- vs. EDTA-isolated cells. These data showed that K3EDTA blood samples resulted in higher purity of bovine granulocytes compared to lithium heparin blood.

Regarding molecular characteristics, previous reports have described the potential of heparin for endotoxin-induced tumor necrosis factor-alpha production in human monocytes (Heinzelmann et al., 1999). On the other hand, EDTA seems to inhibit the production of LPS-induced cytokines (Engstad et al., 1997). Furthermore, the detection of membrane-bound LPSb on monocytes differed in EDTA- or heparin-treated blood, and cell activation was better obtained in heparinized blood (Brunialti et al., 2002). Although other reports have described the effects of anticoagulants on clinical assay results, the impact of BM collection tube components has not been well documented in the context of the outcome of the isolation method for subsequent analysis. Therefore, it is important to investigate how anticoagulants in the hBMSC isolation procedure affect cellular and molecular characteristics of stem cells.

Previous studies reported that heparin promotes WNT and FGF signaling and sequestering growth factors in hESCs and MSCs supporting their proliferation (Furue et al., 2008; Sasaki et al., 2008; Ling et al., 2016; Wijesinghe et al., 2017), which goes along with our data on higher number of CFU-f and higher yield of hBMSCs obtained from heparin- vs. EDTA-coated tubes. However, we observed no significant changes in the short-term proliferation assay. In terms of differentiation capacity, heparin has been shown to stimulate OB differentiation of stem cells (Ling et al., 2010), while our results did not show any profound differences between heparin- and EDTA-isolated hBMSCs in terms of OB and AD differentiation. Similar results were reported in the study by Roger et al. (2020) and Laner-Plamberger et al. (2019) using a different source of human stem cells. Previous *in vitro* studies using heparin as a supplement in the expansion media provided promising results to improve the longevity of MSCs and reduce senescence during passaging of the cells (Ling et al., 2016; Samsonraj et al., 2017; Wijesinghe et al., 2017). However, using heparin as an anticoagulant in the hBMSC isolation procedure did not have a big impact on hBMSCs compared to EDTA-isolated cells.

Furthermore, RNA-seq analysis did not show any significant impact of the used anticoagulant on the transcriptome of non-cultivated and cultivated hBMSCs, while *in vitro* cultivation revealed a more significant effect on transcriptomic changes in hBMSCs regardless of the used anticoagulants. As the number of freshly isolated BMSCs is low, their thorough characterization using functional and molecular analyses is limited (Boquest et al., 2005). Previous studies indicated that cell culture conditions affect the phenotypic characteristics of BMSCs, but only a few findings document these changes (Ben Azouna et al., 2012; Hagmann et al., 2013; Kim et al., 2018; Tan et al., 2022). In our study, GEO analysis identified genes that exhibited higher expression with cultivation compared to non-cultivated cells. These genes were involved in the ribonucleotide metabolic process (COX14, NDUFB5—NADH:ubiquinone oxidoreductase subunit B5; LDHA—lactate dehydrogenase A; and PAPSS2—3′-phosphoadenosine 5′-phosphosulfate synthase 2) and generation of precursor metabolites and energy. Resetting pluripotency through nuclear reprogramming and redirecting stem cells into defined lineages underlines exceptional cell fate plasticity. Modulation of energy metabolism associated with mitochondrial biogenesis and the maturation of efficient oxidative ATP generation is associated with cell identity determination, which changes bioenergetic demands of stem cells (Folmes et al., 2012). The vital function of bioenergetics in regulating stemness and lineage specification implicates a wider role for metabolic reprogramming in cell fate decision (Folmes et al., 2012), which corresponds with our RNA-seq data on a GEO-enriched subset of genes involved in the mitochondrial respiratory chain complex assembly and mitochondrial gene expression (e.g., TIMMDC1, UQCC1, COX20, and NDUFC2), which are expressed in cultivated hBMSCs. The genes that were downregulated with the *in vitro* cultivation were related to decreased inflammatory properties of the cells, decreased inhibitory factors on the cell cycle, and chromatin remodeling suggesting the maintenance of stable expression states of transcription factor genes (TFDP2 and RBL2—RB transcriptional corepressor-like 2), the control of stem cell activity, higher proliferation, and the active metabolic status of the stem cells. Thus, our findings point out that the bigger difference in the preparation of hBMSCs is caused by the cultivation *in vitro* than the use of different anticoagulants during BM collection. However, using bulk RNA-seq data cannot give detailed information about the heterogeneity of hBMSCs obtained from different isolation protocols. Therefore, future studies should take this into consideration for protocol validation as *in vitro* cultivation may prefer expanding specific cell types, which can be overlooked by functional assays without cell selection (Hoover et al., 2023). Moreover, the molecular analyses of BMSC quality *in vitro*, it is also important to test stem cell potential using *in vivo* experiments depending on the context of the clinical studies (Robey et al., 2014).

Furthermore, metabolomic analysis in human BM plasma obtained from paired donor samples in heparin- and EDTA-coated tubes revealed only minor changes showing the enrichment of tricarboxylic acid cycle metabolites in EDTA compared to heparin BM plasma. The increase in *N*-acetyl forms of some amino acids and dipeptides in heparin samples can be explained as artifacts originating from enzymatic reactions with corresponding precursors present in the diluting medium. This is the first time we have investigated the impact of anticoagulants on metabolome and lipidome in human BM plasma. Previous metabolomic analyses in human plasma samples revealed changes in amino acid levels, and lipids in EDTA-coated tubes were explained by the effect on ionization efficiency in differently prepared blood samples (Barri and Dragsted, 2013; Khadka et al., 2019; Sotelo-Orozco et al., 2021). Therefore, it is important to record the information about the BM and plasma sample collection in relation to further subsequent molecular analyses in collected samples and comparing the data among different clinical centers.

The present study has several positive aspects. First, it has a unique study design with a paired sample from the same donor for comparison analysis, which has not been performed before. Furthermore, we employed state-of-the-art methods to characterize hBMSC cellular and molecular properties with a broad spectrum of different molecular methods including differentiation and functional assays, metabolomics, and RNA-seq. In addition, it analyzes the relationship between cultured and freshly isolated hBMSCs in relation to their molecular changes and heterogeneity.

On the other hand, our study has some limitations. It includes a small number of samples (only 12, 6M/6F) and duplicates in specific assays used in the analysis, which would need to be extended in future studies. Furthermore, the analysis should be performed in another cohort of healthy donors to confirm the outcome of the study as isolated hBMSC samples were obtained from patients with cancer. Moreover, using the single-cell RNA-seq analysis would better unveil the heterogeneity of isolated hBMSCs obtained from different isolation methods.

## Conclusion

Our study using paired samples of hBMSCs from heparin- and EDTA-coated tubes showed that the type of anticoagulant during BM collection has a minimum effect on molecular characteristics of hBMSCs, while *in vitro* cultivation has a major impact on the transcriptomic profile of isolated cells. Moreover, our data provide insight into the importance of cellular quality depending on the isolation and cultivation conditions. More importantly, these findings shed light on the preparation of hBMSCs for future clinical use and minimize the heterogeneity in the isolation procedures. Future studies should be followed, including testing in multiple clinical centers.

## Data Availability

The data that support the findings of this study are available on the request from the corresponding author. The raw data of RNA sequencing from the patient samples are available on the public repository https://doi.org/10.5281/zenodo.7936622.

## Abbreviations

BM, bone marrow; CFU-f, colony-forming unit-fibroblasts; DEGs, differentially expressed genes; ECAR, extracellular acidification rate; EDTA, ethylenediaminetetraacetic acid; FCCP, carbonyl cyanide-4-(trifluoromethoxy) phenylhydrazone; hBMSCs, human bone marrow mesenchymal stem cells; MSCs, mesenchymal stem cells; OCR, oxygen consumption rate; ORA, overrepresentation analysis; PCA, principal component analysis.
