# Supplementary material for: Human bone marrow stromal cells: the impact of anticoagulants on stem cell properties
Source: Front Cell Dev Biol. 2023 Sep 18;11:1255823. doi: 10.3389/fcell.2023.1255823 (PMC10544901; doi:10.3389/fcell.2023.1255823)
Supplement: Supplementary file 1 [file DataSheet1.pdf]

SUPPLEMENTARY MATERIAL

CONTENT:

Supplemental Tables: Supplemental Table 1-4

Supplemental Figures with figure legends: Supplemental Figure 1-5

SUPPLEMENTAL TABLES

Supplemental Table 1. List of primer sequences used for RT-qPCR.

Gene	Forward sequence	Reverse sequence
β-ACTIN	ATTGGCAATGAGCGGTTCCG	AGGGCAGTGATCTCCTTCTG
RUNX2	TGGTTACTGTCATGGCGGGTA	TCTCAGATCGTTGAACCTTGCTA
BGLAP	CACTCCTCGCCCTATTGGC	CCCTCCTGCTTGGACACAAAG
COL1A1	GAGGGCCAAGACGAAGACATC	CAGATCACGTCATCGCACAAC
PPARγ	CTCCTATTGACCCAGAAAGCGA	TGCCATGAGGGAGTTGGAAG
CEBPA	AACCTTGTGCCTTGGAAATG	CTGTAGCCTCGGGAAGGAG
ADIPOQ	GGGCCCCAGGCCGTGATGGCA	TCGGGGACCTTCAGCCCCGGGTA
FABP4	TGGGGGTGTCCTGGTACATGTGCAGAAAT	ACGCCTTTCATGACGCATTCCACCACC

Supplemental Table 2. List of fluorescent conjugated antibodies used for flow cytometry.

Stem cell marker	Conjugate	Reference	Company
**ALPL**	APC	RD-FAB1448A	R&D System
**LepR/CD295**	FITC	RD-FAB867F	R&D System
**CD44**	PE	BZ-338808	Biolegend
**CD73**	PE	BZ-344004	Biolegend

Supplemental Table 3. Metabolomic and lipidomic data of BM plasma of patients from paired samples of Hepa and EDTA-coated tubes.

Supplemental Table 4. RNA seq data from non-cultivated and cultivated hBMSCs.

Supplemental Figure 1.

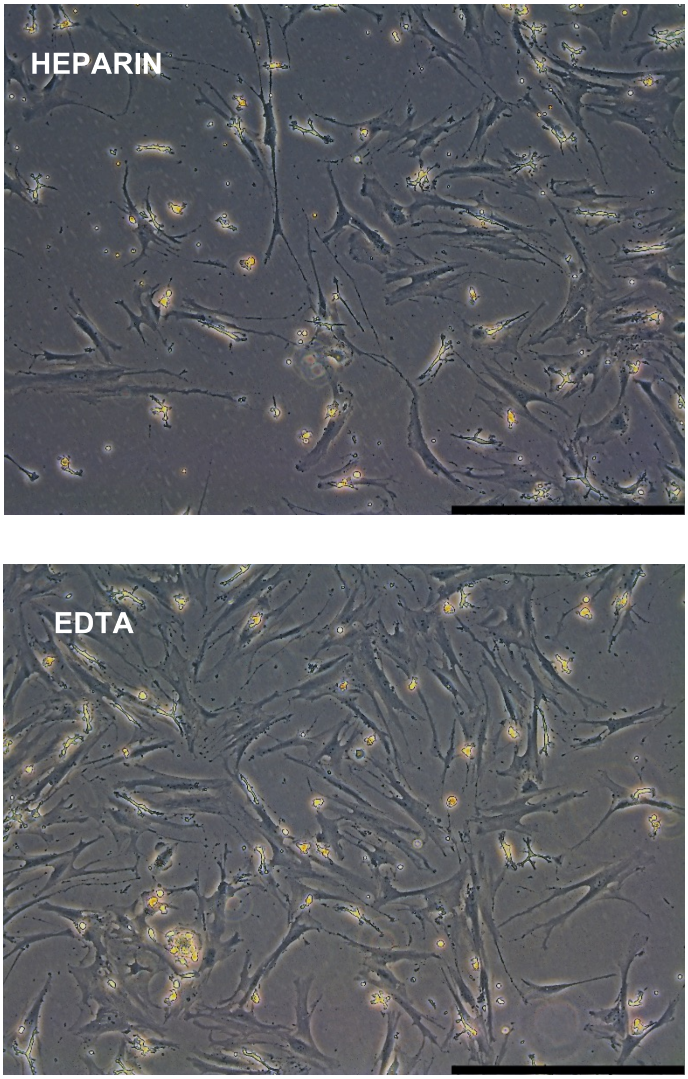

Supplemental Figure 1 legend: (A) Enlarged pictures of Figure 1H.

Supplemental Figure 2.

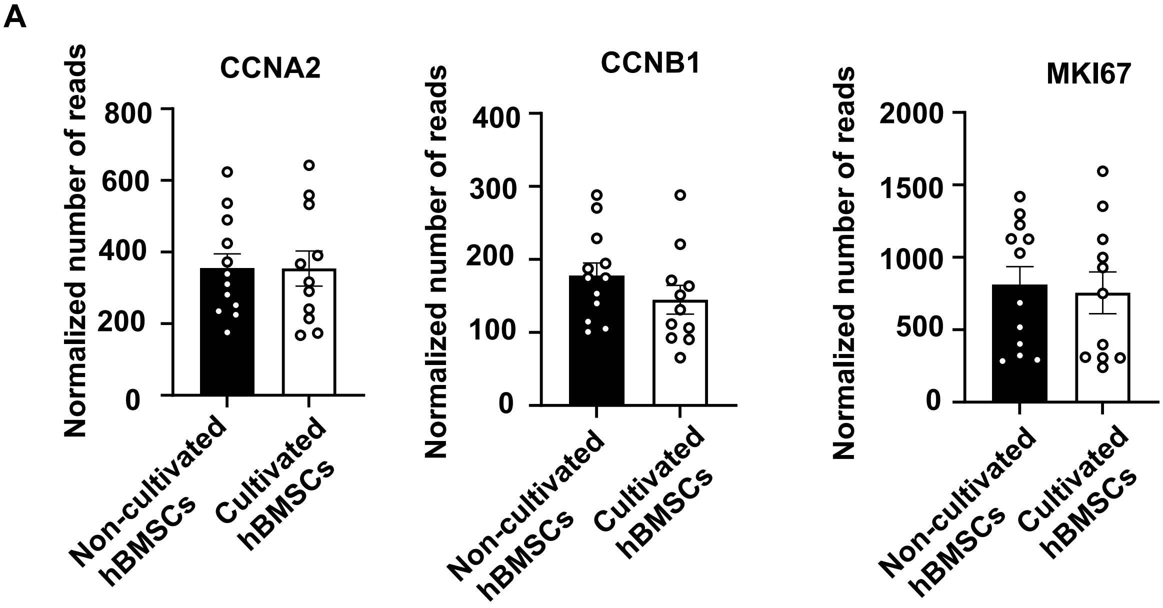

Supplemental Figure 2 legend: Data from RNA-seq analysis of proliferation markers showing effect of cultivation on hBMSCs. (A) Gene expression profile of proliferation markers using RNA seq analysis in non-cultivated and cultivated hBMSCs from two selected donors. Data are presented as normalized number of reads; (n = 2; 11-12 replicates per sample; $p > 0.05$, paired Student's t-test).

Supplemental Figure 3.

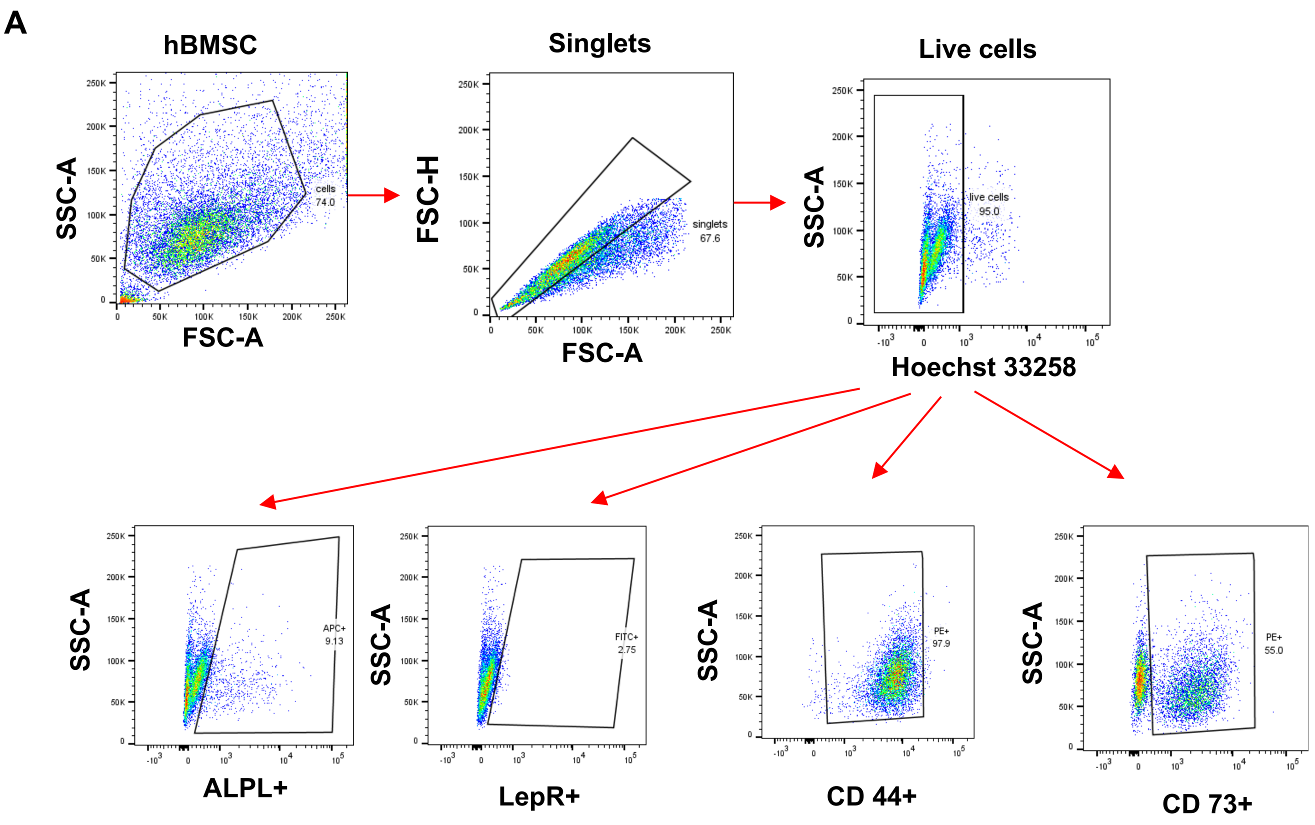

Supplemental Figure 3 legend: (A) Flow cytometry gating strategy for immunophenotyping of hBMSCs.

Supplemental Figure 4.

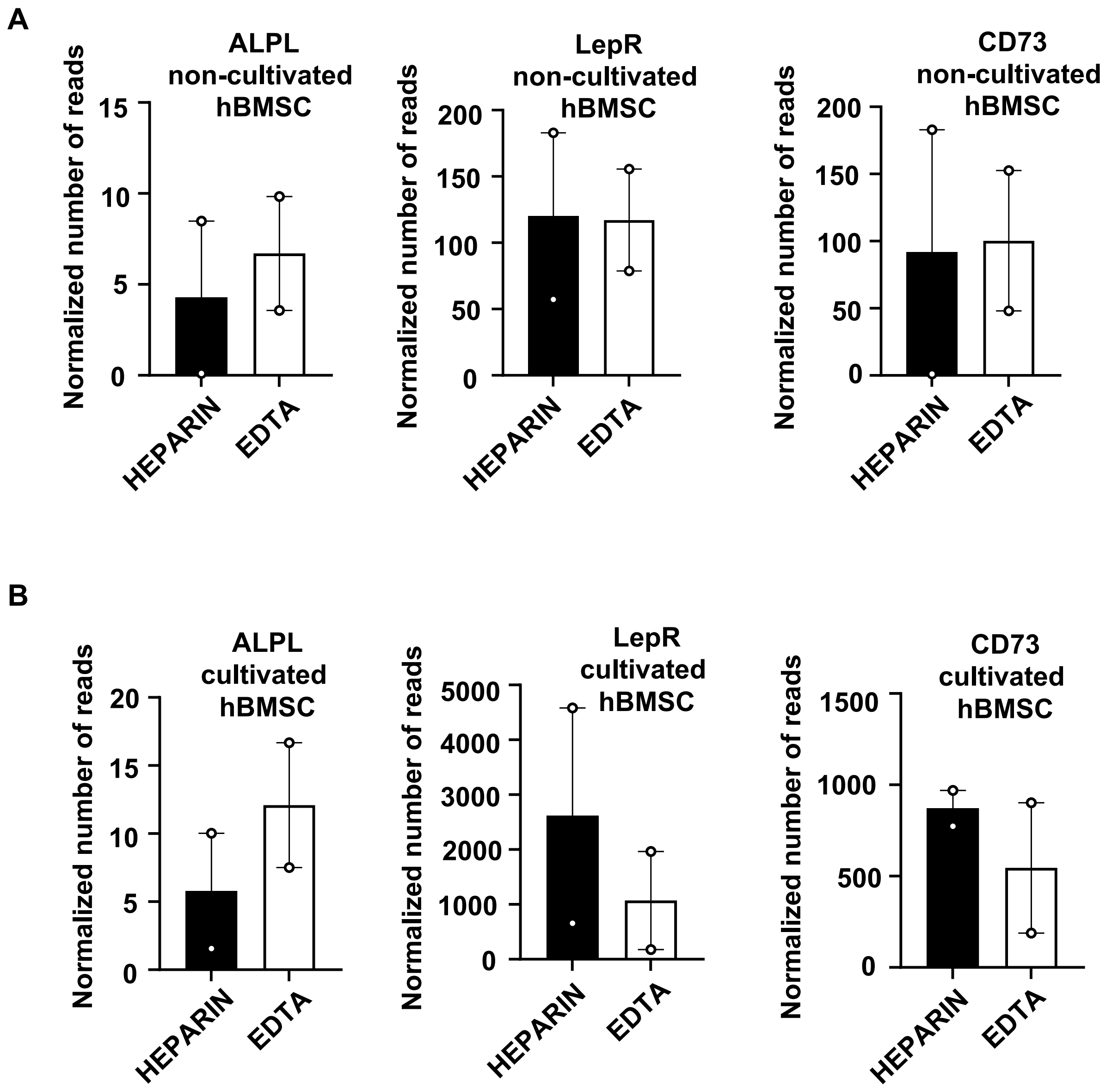

Supplemental Figure 4 legend: Data from RNA-seq analysis confirming flow cytometry results of the effect on the cell surface markers of non-cultivated and cultivated hBMSCs. (A) Gene expression profile of stem cell surface markers using RNA seq analysis in non-cultivated hBMSCs obtained from Heparin-coated and EDTA-coated tubes from two selected donors. Data are presented as normalized number of reads; ($n = 2$; 3 replicates per sample; $p > 0.05$, paired Student's t-test). **(B)** Gene expression profile of stem cell surface markers using RNA seq analysis in cultivated hBMSCs obtained from Heparin-coated and EDTA-coated tubes from two selected donors. Data are presented as normalized number of reads; ($n = 2$; 3 replicates per sample; $p > 0.05$, paired Student's t-test).

Supplemental Figure 5.

A

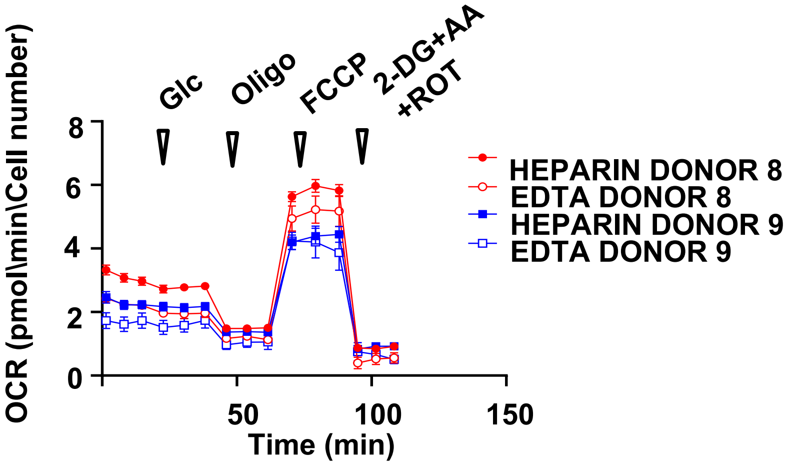

B

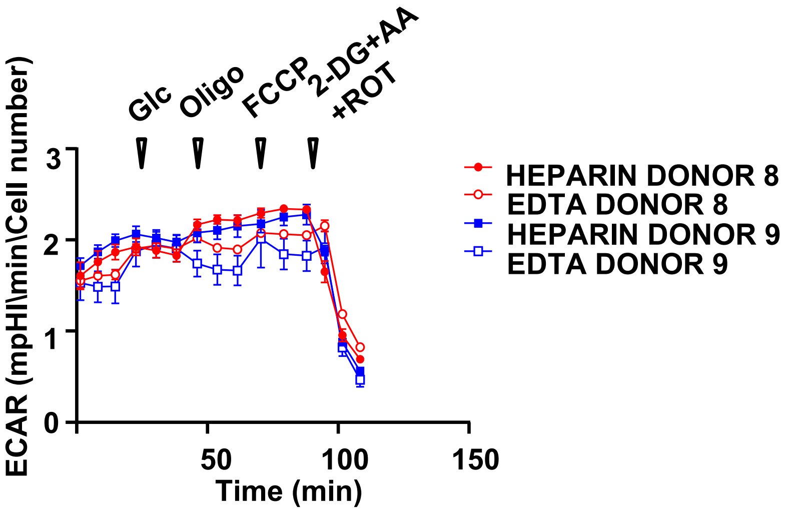

Supplemental Figure 5 legend: The effect of Heparin and EDTA anticoagulants on bioenergetic profile of hBMSCs. (**A**) Measurement of OCR of primary hBMSCs isolated from Heparin and EDTA-coated tubes; Data are presented as mean ± SEM (n = 2 independent experiments with five replicates per sample). (**B**) Measurement of ECAR of hBMSCs isolated from Heparin and EDTA-coated tubes; Data are presented as mean ± SEM (n = 2 independent experiments with five replicates per sample).
